# Some Points in the Diagnosis and Treatment of Dysentery Occurring in the British Salonika Force

**Published:** 1918-05

**Authors:** 


***Some Points in the Diagnosis and Treatment of Dysentery Occurring in the British Salonika Force.*** By DUNCAN GRAHAM, M. B. (Toronto), Temporary Major, C. A. M. C. Abs. from the *Lancet*, Jan. 12, 1918.

Among 2500 cases of dysentery investigated from June 1916 up to the present time 3 per cent. or less were amebic and the rest bacillary. All the amebic cases were treated by emetine, and some who continued to pass cysts in their faeces were given a course of double iodide of bismuth and emetine with resultant disappearance of the cysts in 48 hours.

Of the severe and moderately severe bacillary cases, bacteriological examination showed that 75 per cent. were due to B. dysenteriae Shiga and the remainder to B. dysenteriae Flexner-Y. 50 per cent of the fatal cases were B. dysenteriae Flexner Y.

Of the mild cases 75 per cent. were Flexner-Y and the remainder Shiga.

Mild recurrent cases were practically all due to Flexner-Y.

The comparatively few cases of chronic bacillary dysentery were due to be B. dysenteriae Shiga, though less severe cases due to Flexner-Y were seen.

The author's observations go to show that flagellates never produce dysentery and that it is very doubtful whether they can be regarded as causal agents in simple diarrhea. The prevention of dehydration of the tissues is of great importance. Intravenous injections of antidysenteric serum in doses of 60 to 80 cc., followed by from 150 to 300 cc. of normal saline, once or twice daily during the first 3 days of treatment, give good results.

